# Association between the side effect induced by COVID-19 vaccines and the immune regulatory gene polymorphism

**DOI:** 10.3389/fimmu.2022.941497

**Published:** 2022-10-26

**Authors:** Ding-Ping Chen, Ying-Hao Wen, Wei-Tzu Lin, Fang-Ping Hsu

**Affiliations:** ^1^ Department of Laboratory Medicine, Linkou Chang Gung Memorial Hospital, Taoyuan, Taiwan; ^2^ Department of Medical Biotechnology and Laboratory Science, College of Medicine, Chang Gung University, Taoyuan, Taiwan; ^3^ Graduate Institute of Biomedical Sciences, College of Medicine, Chang Gung University, Taoyuan, Taiwan; ^4^ School of Medicine, National Tsing Hua University, Hsinchu, Taiwan; ^5^ School of Medicine, Chang Gung University, Taoyuan, Taiwan; ^6^ Graduate Institute of Clinical Medical Sciences, College of Medicine, Chang Gung University, Taoyuan, Taiwan

**Keywords:** single nucleotide polymorphism (SNP), immune regulatory genes, COVID-19, vaccine, side effect

## Abstract

People often worry about the side effects after vaccination, reducing the willingness to vaccinate. Thus, we tried to find out the risk of single nucleotide polymorphism (SNP) vaccines to improve the willingness and confidence in vaccination. Allergic and inflammatory reactions are the common vaccine side effects caused by immune system overreaction. In addition, a previous study showed significantly higher frequency of febrile reactions to measles vaccines in American Indians than in Caucasian children, indicating that the side effects varied in accordance with genetic polymorphisms in individuals. Thus, SNPs of immune regulatory genes, cytotoxic T-lymphocyte-associated protein 4 (CTLA4), CD28, tumor necrosis factor ligand superfamily member 4 (TNFSF4) and programmed cell death protein 1 (PDCD1) were included in this study to analyze their association with vaccine side effects. Moreover, 61 healthy participants were asked on the number of doses they received, the brand of the vaccine, and the side effects they suffered. We found that several SNPs were associated with side effects after the first or second dose of mRNA or adenoviral vector vaccines. Furthermore, these SNPs were associated with several autoimmune diseases and cancer types; thus, they played an important role in immune regulation. Moreover, rs3181096 and rs3181098 of CD28, rs733618 and rs3087243 of CTLA, and rs1234314 of TNFSF4 were associated with mild vaccine side effects induced by mRNA and adenoviral vector vaccines, which would play a potential role in vaccine-induced immune responses and may further lead to fatal side effects. These results could serve as a basis for investigating the mechanism of vaccine side effects. Furthermore, it was hoped that these results would address public concerns about the side effects of the COVID-19 vaccination. In clinical application, a rapid screening test can be performed to assess the risk of vaccine side effects before vaccination and provide immediate treatment.

## Introduction

Recently, COVID-19 has caused a pandemic of severe acute respiratory syndrome (SARS-CoV-2). At present, the policies of various countries are inclined to coexist with the virus; thus, the vaccination rate must be improved. Although vaccination is a safe way of protecting people against infection and severe complications, people still worry about the side effects of vaccination. In addition, several studies have shown that the host genotype has a great effect on the immune response induced by vaccines, such as influenza, hepatitis B, and measles vaccination (1–3). Therefore, identifying single nucleotide polymorphisms (SNPs) that increase the risk of side effects of COVID-19 vaccines could improve people’s willingness and confidence in vaccination through genetic testing.

Vaccines are designed to stimulate antigen-specific T cell responses and enhance adaptive immunity. However, excessive T cell response may cause vaccine-induced side effects. Although the process of vaccine production and development have undergone rigorous testing, varying severities of side effects will be reported after large-scale vaccination. The most common side effects of COVID-19 vaccines are mild, such as soreness, swelling or redness at the injection site, fever, rash, and pain. Although the serious side effects are rare, they may cause seizures, thrombosis, myocarditis, or life-threatening allergic reactions (4). Fever is the most common vaccine side effect, which can occur after any vaccine is given. Fever is defined as a core body temperature above 38°C. In addition, the common mechanism of fever is induced by tumor necrosis factor (TNF), which increases the secretion of interleukin (IL)-1 and IL-6, and acts on the hypothalamus to increase body temperature. This mechanism will increase the activity and mobility of leukocytes, stimulate the production of interferon, and activate T cells to enhance immune response (5). The immune checkpoints involved in T cell activation, PDCD1 and CTLA4 are associated with the mechanism of fever (6).Moreover, Tritto et al. (7) hypothesized that the injection-site reaction was due to the use of adjuvants. The adjuvant provides the second signal that is necessary for T cell activation and increases the release of cytokines and chemokines; hence, immune cells in the blood will gather at the injection site, leading to redness, swelling and pain (8). Furthermore, headache is a common side effect after inoculating viral vaccines. The frequency of headaches was 39% to 80% after mRNA vaccination (9, 10) and from 39% to 66% after adenoviral vector vaccination (11). Headache is due to calcitonin gene-related peptide, an inflammatory neuropeptide that stimulates the secretion of TNFα, IL-1β and IL-6 when combined with receptors on T cells, which induces the release of neurogenic inflammation and vasodilation in pial vessels (12, 13). Notably, the risk of side effects after giving the same vaccine varies between groups and individuals. This phenomenon was first observed by the team of Black et al. They observed a significantly higher frequency of febrile reactions to measles vaccines in American Indians than in Caucasian children (14), suggesting that genes play a role in the occurrence of side effects after vaccination. The immune response induced by the vaccine was also related to genetic factors based on a twin study (15), which shows that the immune response after vaccination can be predicted by an individual genotype.

In literature, no studies have investigated genotypes associated with the side effects of COVID-19 vaccines. However, SNPs susceptible to disease risk or disease severity may be involved in the immune response of vaccination, leading to vaccine-induced adverse reactions (16). Therefore, this study conducted a detailed analysis to investigate the association between genes and the second signal for T cell activation and understand the effect of genes on the side effects after vaccination, including cytotoxic T-lymphocyte-associated protein 4 (CTLA4), CD28, tumor necrosis factor ligand superfamily member 4 (TNFSF4), and programmed cell death protein 1 (PDCD1) and the side effects caused by COVID-19 vaccination.

## Materials and methods

### Subjects

In this study, 61 healthy adults who received two doses of COVID-19 vaccine were enrolled from Chang Gung Memorial Hospital (CGMH, Taoyuan, Taiwan). Amongst them, 24 received two doses of mRNA-1273 (Moderna), 24 received two doses of ChAdOx1-S (Oxford/AstraZeneca, AZ), 5 received two doses of BNT162b2 (Pfizer/BioNTech, BNT), 5 received MVC-COV1901 (Medigen), 2 received one dose of AZ followed by one dose of Moderna, and 1 received one dose of Moderna followed by one dose of BNT. Participants were asked on the number of doses they received, the brand of the vaccine and the side effects they experienced, including fever, pain, redness, or swelling at the injection site, shivering, nausea/anorexia, vomiting, diarrhoea, headache, fatigue, muscle pain, joint pain, and skin allergy. All data were self-reported. The study has been reviewed and approved by the Institutional Review Board of CGMH. The approved ID was 202101837B0. All the participants have signed the consent form.

### DNA extraction

We collected oral mucosal cells of these participants to extract genomic DNA using the QIAamp DNA Mini Kit (Qiagen, Germany). Then, the purity and concentration of the extracted DNA were determined by measuring the absorbance at 260 and 280 nm. Then, the extracted DNA were stored for the following experiments.

### PCR and SNP analysis

A total of four costimulatory genes (CTLA4, TNFSF4, CD28, and PDCD1) were investigated in this study. We focused on the promoter region of genes, because the SNP located in the promoter region may affect the expression level of gene (17). In addition, the hot points of autoimmune diseases and cancer types were considered, including rs231775 in exon1 of CTLA4 (18, 19), rs3087243 in 3 prime untranslated region (3’UTR) of CTLA4 (19) and rs11568821 in intron4 of PDCD1 (20). Thus, eight fragments were selected for SNP analysis. The pairs of primers are shown in **Table 1**. These eight amplified DNA fragments consisted of 44 candidate SNPs (**Table 2**). The PCR volume was 25μL, including each 1μL of forward and reverse primer, 8μL of Hot Start Taq DNA polymerase (Agilent, Santa Clara, California, USA), 1μL of sample DNA and 14μL of ddH2O. The PCR programme was initiated as follows: 4 min at 94°C, 30 cycles of 30 s at 94°C, 30 s at 58°C, 45 s at 72° C and final 10 min at 72°C. Subsequently, the 5μL of PCR products was fractionated on the 1.5%-2% agarose gel. According to the manufacturer’s instructions, the Big Dye Terminator Cycle Sequencing kit (Thermo Fisher, Waltham, Massachusetts, USA) and ABI PRISM genetic analyser (Thermo Fisher, Waltham, Massachusetts, USA) were used for direct sequencing. Given the insufficient genomic DNA and failure of PCR, complete SNP data were not available.

**Table 1:** The pairs of primer for amplification of candidate SNPs.

non-HLA gene	Primer
TNFSF4-Promoter	5’ GGCTT GGA GTC TAT GAT ATT GTG CC 3’ 5’ GAA GGG CGT TTA ACC ACA CTT TAC G 3’
CD28-Promoter	5’- GGG TGG TAA GAA TGT GGA TGA ATC-3’ 5’-CAA GGC ATC CTG ACT GCA GCA-3’
CTLA4-Promoter 1	5’ GGC AAC AGA GAC CCC ACC GTT 3’ 5’ GAG GAC CTT CCT TAA ATC TGG AGA G 3’
CTLA4-Promoter 2	5’CTC TCC AGA TTT AAG GAA GGT CCT C 3’ 5’GGA ATA CAG AGC CAG CCA AGC C3’
CTLA4-Exon4	5’ CTA GGG ACC CAA TAT GTG TTG 3’ 5’ AGA AAC ATC CCA GCT CTG TC 3’
CTLA4-3UTR	5’GCT TGG AAA CTG GAT GAG GTC ATA GC 3’ 5’AGA GGA AGA GAC ACA GAC AGA GTT GC 3’
PDCD1-Promoter	5’-ACCCACACAGCCTCACATCTCT-3’5’-AAA CTG AGG GTG GAA GGT CCC T-3’
PDCD1-Intron4	5’-TGGTGACCCCAAGTGTGTTTCTC-3’ 5’-GAG GAA TTT TTC ACC GGA GGG C-3’

**Table 2:** The 44 candidate SNPs that were analyzed the association with vaccine-induced reaction.

Gene	SNP under analysis				
TNFSF4	rs181758110	rs45454293	rs1234314	rs147669352	
CD28	rs1879877	rs3181096	rs3181097	rs3181098	rs28718975
	rs28688913	rs28541784	rs201801072	rs200353921	
CTLA4	rs11571315	rs733618	rs4553808	rs11571316	rs62182595
	rs573554201	rs16840252	rs945677329	rs5742909	rs231775
	rs56102377	rs56217811	rs1581575882	rs980967681	rs55696217
	rs231721	rs778932058	rs3087243	rs11571319	
PDCD1	rs10204525	rs1331108508	rs56029561	rs2227981	rs2227982
	rs6705653	rs41386349	rs11568821	rs36084323	rs1044067342
	rs5839828	rs944761632			

### Statistical analysis

The Chi-square test and Fisher’s exact test were used to analyze the association between the vaccine-induced adverse reaction and the genotype frequency of participants using SPSS17.0. All data were analyzed through the dominant (AA vs. Aa+aa), recessive (AA+Aa vs. aa), and additive (AA vs. Aa vs. aa) models. Significant SNPs are summarized in **Tables 3**–**6**.

**Table 3:** Side effects with the first dose of mRNA vaccines (Moderna +BNT).

SNP	Gene position	No. of patients (%)			Model	Logistic regression *P*	Odds ratio	95% Confidence Interval	
								Lower	Upper
Fever									
rs56029561	PDCD1	CAG	CAG/del	del	Additive	0.010	–	–	–
Yes	3’UTR	0	3	4	CAG/del+del vs CAG	–	–	–	–
No		0	0	22	CAG/del+CAG *vs* del	0.010	–	–	–
Injection site									
rs3181098	CD28	GG	AG	AA	Additive	0.016	–	–	–
Yes	promoter	19	4	4	AG+AA vs. GG	0.111	–	–	–
No		0	2	0	GG+AG vs. AA	1.000	–	–	–
Chills									
rs733618	CTLA4	CC	CT	TT	Additive	0.026	–	–	–
Yes	promoter	1	6	1	CT+TT vs. CC	1.000	0.737	0.057	9.457
No		2	5	14	CT+CC vs. TT	0.014	0.071	0.007	0.701
rs3181096	CD28	CC	CT	TT	Additive	0.025	–	–	–
Yes	promoter	3	5	0	CT+TT vs. CC	0.406	0.369	0.069	1.982
No		13	3	5	CT+CC vs. TT	0.283	–	–	–
rs2227982	PDCD1	AA	AG	GG	Additive	0.039	–	–	–
Yes	exon 5	6	2	0	GG+AG vs. AA	0.030	9.000	1.355	59.783
No		5	10	5	AG+AA vs. GG	0.281	–	–	–
rs36084323	PDCD1	TT	CT	CC	Additive	0.064	–	–	–
Yes	promoter	6	1	0	CT+CC vs. TT	0.033	11.143	1.108	112.012
No		7	9	4	CT+TT vs. CC	0.545	–	–	–
Diarrhea									
rs5839828	PDCD1	del/del	G/del	GG	Additive	0.046	–	–	–
Yes	promoter	3	2	2	del/G+delvs. GG	0.060	–	–	–
No		12	8	0	del/G+GG vs. del	0.662	0.500	0.087	2.860
Headache									
rs2227982	PDCD1	AA	AG	GG	Additive	0.026	–	–	–
Yes	exon 5	8	6	0	GG+AG vs. AA	0.053	4.889	0.931	25.670
No		3	6	5	AG+AA vs. GG	0.041	–	–	–
rs36084323	PDCD1	TT	CT	CC	Additive	0.080	–	–	–
Yes	promoter	8	6	0	CT+CC vs.. TT	0.332	0.469	0.101	2.185
No		5	4	4	CT+TT vs.. CC	0.041	1.444	1.005	2.075
Myalgia									
rs1234314	TNFSF4	CC	CG	GG	Additive	0.003	–	–	–
Yes	promoter	3	7	0	CG+CC vs.. GG	0.005	–	–	–
No		0	9	10	CG+GG vs..CC	0.033	–	–	–
rs2227982	PDCD1	AA	AG	GG	Additive	0.046	–	–	–
Yes	exon 5	7	2	1	AG+AA vs.. GG	0.626	2.571	0.246	26.851
No		4	10	4	GG+AG vs.. AA	0.020	8.167	1.419	47.016
rs36084323	PDCD1	TT	CT	CC	Additive	0.081	–	–	–
Yes	promoter	7	1	1	CT+CC vs.. TT	0.046	7.000	1.098	44.608
No		6	9	3	CT+TT vs.. CC	1.000	1.600	0.142	18.000
Skin allergy									
rs3181096	CD28	CC	CT	TT	Additive	0.086	–	–	–
Yes	promoter	5	0	0	CT+TT vs.. CC	0.048	–	–	–
No		11	8	5	CT+CC vs.. TT	0.553	–	–	–
rs200353921	CD28	TT	AT	AA	Additive	0.064			
Yes	promoter	2	0	3	AT+AA vs.. TT	0.123	0.185	0.024	1.432
No		18	2	3	TT+AT vs.. AA	0.050	0.100	0.012	0.869

Additive: AA vs. Aa vs. aa.

**Table 4:** Side effects with the second dose of mRNA vaccine (Moderna +BNT).

SNP	Gene position	No. of patients (%)			Model	Logistic regression *P*	Odds ratio	95% Confidence Interval	
								Lower	Upper
Fever									
rs2227982	PDCD1	AA	AG	GG	Additive	0.030	–	–	–
Yes	exon 5	7	8	0	GG+AG vs. AA	0.390	1.969	0.416	9.317
No		4	4	5	AG+AA vs. GG	0.013	0.348	0.199	0.609
rs36084323	PDCD1	CC	CT	TT	Additive	0.022			
Yes	promoter	0	8	8	CT+CC vs. TT	0.816	1.200	0.257	5.593
No		4	2	5	CT+TT vs. CC	0.019	–	–	–
Injection site									
rs11571315	CTLA4	CC	CT	TT	Additive	0.039	–	–	–
Yes	promoter	3	14	11	CT+TT vs. CC	0.138	0.107	0.037	0.312
No		1	0	0	CT+CC vs. TT	1.000	0.607	0.451	0.818
rs231775	CTLA4	AA	AG	GG	Additive	0.016	–	–	–
Yes	exon1	2	13	11	AG+AA vs. GG	1.000	0.577	0.415	0.802
No		1	0	0	GG+AG vs. AA	0.111	0.077	0.020	0.291
Chills									
rs3181096	CD28	CC	CT	TT	Additive	0.051	–	–	–
Yes	promote	4	6	3	CT+TT vs. CC	0.017	0.148	0.029	0.759
No		12	2	2	CT+CC vs. TT	0.632	0.476	0.067	3.396
rs11571316	CTLA4	AA	AG	GG	Additive	0.121	–	–	–
Yes	promoter	1	7	5	AG+AA vs. GG	0.047	4.800	0.979	23.544
No		1	3	12	GG+AG vs. AA	1.000	1.250	0.071	22.132
rs5839828	PDCD1	del	del/G	GG	Additive	0.046	–	–	–
Yes	promoter	9	2	2	del/G+del vs. GG	0.222	–	–	–
No		6	8	0	del/G+GG vs. del	0.168	3.000	0.616	14.617
Headache									
rs3087243	CTLA4	GG	AG	AA	Additive	0.014	–	–	–
Yes	3UTR	6	8	2	AG+AA vs. GG	0.006	0.429	0.234	0.785
No		8	0	0	GG+AG vs. AA	0.536	–	–	–
rs3181097	CD28	GG	AG	AA	Additive	0.021	–	–	–
Yes	promote	10	5	2	AG+AA vs. GG	0.008	15.714	1.634	151.125
No		1	7	4	GG+AG vs. AA	0.198	3.750	0.560	25.121
rs3181098	CD28	GG	AG	AA	Additive	0.039	–	–	–
Yes	promote	8	5	4	AG+AA vs. GG	0.019	0.081	0.008	0.773
No		11	1	0	GG+AG vs. AA	0.121	–	–	–
Myalgia									
rs3181097	CD28	AA	AG	GG	Additive	0.021	–	–	–
Yes	promoter	2	5	10	AG+AA vs. GG	0.008	0.064	0.007	0.612
No		4	7	1	GG+AG vs. AA	0.198	0.267	0.040	1.786
rs231775	CTLA4	AA	AG	GG	Additive	0.035	–	–	–
Yes	exon1	1	11	4	AG+AA vs. GG	0.061	5.250	0.988	27.895
No		2	2	7	GG+AG vs. AA	0.549	0.300	0.024	3.799

Additive: AA vs. Aa vs. aa.

**Table 5:** Side effects with the first dose of adenoviral vector vaccine (AZ).

SNP	Gene position	No. of patients (%)			Model	Logistic regression *P*	Odds ratio	95% Confidence Interval	
								Lower	Upper
Diarrhea									
rs3181096	CD28	CC	CT	TT	Additive	0.004	–	–	–
Yes	promoter	0	2	1	CT+TT vs. CC	0.028	–	–	–
No		16	5	0	CT+CC vs. TT	0.125	–	–	–
rs3181098	CD28	GG	AG	AA	Additive	0.004	–	–	–
Yes	promoter	0	2	1	AG+AA vs. GG	0.028	–	–	–
No		16	5	0	GG+AG vs. AA	0.125	–	–	–
rs1234314	TNFSF4	CC	CG	GG	Additive	0.042	–	–	–
Yes	promoter	2	1	0	CG+GG vs. CC	0.061	0.053	0.003	0.872
No		2	14	5	CG+CC vs. GG	1.000	–	–	–
Headache									
rs1879877	CD28	GG	GT	TT	Additive	0.009	–	–	–
Yes	promoter	2	13	2	GT+TT vs. GG	1.000	0.800	0.061	10.562
No		1	1	5	GG+GT vs. TT	0.009	18.750	2.065	170.215
Skin allergy									
rs733618	CTLA4	CC	CT	TT	Additive	0.026	–	–	–
Yes	promoter	1	0	0	CT+TT vs. CC	0.125	–	–	–
No		2	12	9	CT+CC vs. TT	1.000	–	–	–

Additive: AA vs. Aa vs. aa.

**Table 6:** Side effects with the second dose of adenoviral vector vaccine (AZ).

SNP	Gene position	No. of patients (%)			Model	Logistic regression *P*	Odds ratio	95% Confidence Interval	
								Lower	Upper
Chills									
rs733618	CTLA4	CC	CT	TT	Additive	0.027	–	–	–
Yes	promoter	2	2	0	CT+TT vs. CC	0.061	0.053	0.003	0.872
No		1	10	9	CT+CC vs. TT	0.259	–	–	–
rs980967681	CTLA4	GG	AG	AA	Additive	0.020	–	–	–
Yes	3UTR	4	0	0	AG+AA vs. GG	0.020	–	–	–
No		14	6	0	GG+AG vs. AA	–	–	–	–
Diarrhea									
rs1879877	CD28	GG	GT	TT	Additive	0.026	–	–	–
Yes	promoter	1	0	0	GT+TT vs. GG	0.125	–	–	–
No		2	14	7	GT+GG vs. TT	1.000	–	–	–
rs3181096	CD28	CC	CT	TT	Additive	6E-06	–	–	–
Yes	promoter	0	0	1	CT+CC vs. TT	0.042	–	–	–
No		16	7	0	CT+TT vs. CC	0.333	–	–	–
rs3181098	CD28	AA	AG	GG	Additive	6E-06	–	–	–
Yes	promoter	1	0	0	AG+AA vs. GG	0.333	–	–	–
No		0	7	16	GG+AG vs. AA	0.042	–	–	–
rs3087243	CTLA4	GG	AG	AA	Additive	0.003	–	–	–
Yes	3UTR	0	0	1	AG+AA vs. GG	1.000	–	–	–
No		12	10	1	GG+AG vs. AA	0.083	–	–	–
rs45454293	TNFSF4	GG	AG	AA	Additive	0.003	–	–	–
Yes	promoter	0	0	1	AG+AA vs. GG	0.333	–	–	–
No		16	6	1	GG+AG vs. AA	0.083	–	–	–
rs11571316	CTLA4	AA	AG	GG	Additive	0.003	–	–	–
Yes	promoter	1	0	0	AG+AA vs. GG	1.000	–	–	–
No		1	10	12	GG+AG vs. AA	0.083	–	–	–
Headache									
rs41386349	PDCD1	GG	AG	AA	Additive	0.038	–	–	–
Yes	intron 4	17	4	1	AG+AA vs. GG	0.076	–	–	–
No		0	2	0	GG+AG vs. AA	1.000	–	–	–
Myalgia									
rs733618	CTLA4	CC	CT	TT	Additive	5E-04	–	–	–
Yes	promoter	2	0	0	CT+TT vs. CC	0.011	22.000	3.242	149.298
No		1	12	9	CT+CC vs. TT	0.511	1.692	1.195	2.396
rs3181097	CD28	AA	AG	GG	Additive	0.016	–	–	–
Yes	promoter	2	0	0	AG+AA vs. GG	1.000	1.133	0.953	1.348
No		3	12	7	GG+AG vs. AA	0.036	–	–	–

Additive: AA vs. Aa vs. aa.

## Results

A total of 61 participants who received COVID-19 vaccination were recruited in this study. All participants are Taiwanese. The complete characteristic data of side effects after vaccination in these cases are listed in **Tables S1 and S2**. Most people who took the first dose of the vaccine experienced pain at the injection site (84%), fever (61%), fatigue (62%) and headache (56%) (**Table S1**). Compared with the first dose of vaccination, the frequency of fever, discomfort at the injection site, chill, headache and myalgia was more severe in mRNA vaccines. However, the opposite was true in the adenoviral vector vaccine. Diarrhoea occurred more frequently in the first dose than in the second dose of mRNA vaccines and adenoviral vector vaccines (**Table S2**). The SNP data were obtained to analyze the type of vaccines and the dose number of vaccination, which was divided into the first and second doses of mRNA vaccines (Moderna or BNT) and the first and second doses of adenoviral vector vaccine (AZ).

### Association between SNPs and the side effects of the first dose of mRNA vaccination

Analyzing the side effects induced by the first dose of mRNA vaccines (**Table 3**), rs56029561 of PDCD1 found to be associated with fever (additive, *p*= 0.010; CAG/del +CAG vs. del, *p*=0.010). In addition, rs3181098 of CD28 was associated with discomfort at the injection site (additive, *p*=0.016). rs733618 of CTLA4 (additive, *p*=0.026; CT+CC vs. TT, *p*=0.014, OR=0.071, 95% CI. = 0.007–0.701), rs3181096 of CD28 (additive, *p*=0.025), rs2227982 (additive, *p*=0.039; GG+AG vs. AA, *p*=0.030, OR=9, 95% CI. = 1.355–59.783) and rs36084323 (CT+CC vs. TT, p=0.033, OR=11.143, 95% CI. = 1.108–112.012) of PDCD1 were associated with chills. Moreover, rs5839828 of PDCD1 was associated with diarrhoea based on the additive model (*p*=0.046). rs2227982 (additive, *p*=0.026; AG+AA vs. GG, *p*=0.041, OR=1.444, 95% CI. = 1.005–2.075) and rs36084323 (CT+TT vs. CC, *p*=0.041) of PDCD1 were associated with headache. Furthermore, rs1234314 of TNFSF4 (additive, *p*=0.003; CG+GG vs. CC, *p*=0.033; CG+CC vs. GG, *p*=0.005), as well as rs2227982 (additive, *p*=0.046; GG+AG vs. AA, *p*=0.02, OR=8.167, 95% CI. = 1.419–47.016) and rs36084323 (CT+CC vs. TT, *p*=0.046, OR=7, 95% CI. = 1.098–44.608) of PDCD1, was associated with myalgia. Two SNPs of CD28 were associated with skin allergy, namely, rs3181096 (CT+TT vs. CC, *p*=0.048) and rs200353921 (TT+AT vs. AA, *p*=0.05, OR=0.1, 95% CI. = 0.012–0.869).

### Association between SNPs and the side effects of the second dose of mRNA vaccination

Analyzing the side effects induced by second dose of mRNA vaccines (**Table 4**), two SNPs of PDCD1 were found to be associated with fever, namely, rs2227982 (additive, *p*=0.030; AG+AA vs. GG, *p*=0.013, OR=0.348, 95% CI. = 0.199–0.609) and rs36084323 (additive, *p*=0.022; CT+TT vs. CC, *p*=0.019). In addition, rs11571315 (additive, *p*=0.039) and rs231775 (additive, *p*=0.016) were associated with discomfort at the injection site. rs3181096 of CD28 (CT+TT vs. CC, *p*=0.017, OR=0.148, 95% CI. = 0.029–0.759), rs5839828 of PDCD1 (additive, *p*=0.046) and rs11571316 of CTLA4 (AG+AA vs. GG, *p*=0.047, OR=4.8, 95% CI. = 0.979–23.544) were associated with chills. Moreover, rs3087243 of CTLA4 (Additive, p=0.014; AG+AA vs. GG, p=0.006, OR=0.429, 95% CI. = 0.234-0.785), rs3181097 of CD28 (additive, *p*=0.021; AG+AA vs. GG, *p*=0.008, OR=15.714, 95% CI. = 1634-151.125), and rs3181098 of CD28 (Additive, p=0.039; AG+AA vs. GG, p=0.019, OR=0.081, 95% CI. = 0.008–0.773) were associated with headache. rs231775 (additive, *p*=0.035) of CTLA4 and rs3181097 of CD28 (additive, *p*=0.021; AG+AA vs. GG, *p*=0.008, OR=0.064, 95% CI. = 0.007–0.612) were associated with myalgia.

### Association between SNPs and the side effects of the first dose of adenoviral vector vaccination

Analyzing the side effects induced by the first dose of AZ vaccines (**Table 5**), rs1879877 located in the promoter region of CD28 was found to be associated with headache (additive, *p*=0.009; GG+GT vs. TT, *p*=0.009, OR=18.75, 95% CI. = 2.065–170.215). Three SNPs were associated with diarrhoea, namely, rs3181096 of CD28 (additive, *p*=0.004; AG+AA vs. GG, *p*=0.028), rs3181098 of CD28 (additive, *p*=0.004; AG+AA vs. GG, *p*=0.028), and rs1234314 of PDCD1 (additive, *p*=0.042). Only the rs733618 of CTLA4 was associated with skin allergy (additive, *p*=0.026).

### Association between the SNPs and the side effects of the second dose of adenoviral vector vaccination

Analyzing the side effects induced by the second dose of AZ vaccines (**Table 6**), two SNPs of CTLA4 were found to be associated with chills, namely, rs733618 located in the promoter region (additive, *p*=0.027) and rs980967681 located in the 3’UTR (GG vs. AG, *p*=0.02). In addition, six SNPs were associated with diarrhoea, namely, rs1879877 (GG vs. GT vs. TT, *p*=0.026), rs3181096 (additive, *p*<0.001; CT+CC vs. TT, *p*=0.042; CT+CC vs. TT, *p*=0.042), and rs3181098 (additive, *p*<0.001; GG+AG vs. AA, *p*=0.042) of CD28, rs3087243 (additive, *p*=0.003) and rs11571316 (additive, *p*=0.003) of CTLA4, and rs45454293 of TNFSF4 (additive, *p*=0.003). Moreover, rs41386349 located in the promoter region of PDCD1 was associated with headache (additive, *p*=0.038). rs733618 of CTLA4 (additive, p<0.001; CT+TT vs. CC, *p*=0.011) and rs3181097 of CD28 (additive, *p*=0.016; GG+AA vs. AA, *p*=0.036) were associated with myalgia.

Based on the results, rs3181096 and rs3181098 of CD28, rs733618 and rs3087243 of CTLA4 and rs1234314 of TNFSF4 were associated with side effects induced by mRNA and adenoviral vector vaccines. Furthermore, the data of mRNA and adenovirus vector vaccines were combined for analysis (**Supplementary Tables 3 and 4**). Combining the data of mRNA, subunit, adenoviral vector vaccine, and mixed vaccination, we found that rs733618 and rs3087243 of CTLA4; rs1879877, rs200353921, rs3181096 and rs3181098 of CD28 and the rs10204525, rs2227982, rs2227981, rs6705653, rs41386349 and rs5839828 of PDCD1 were associated with side effects based on the first and second doses of vaccination.

## Discussion

In general, SNPs can be located in coding (exon) or non-coding sequences (intron) of genes (21). If the SNPs are located in the exon region, then they may alter the amino acid sequence of the corresponding protein (missense SNP), introduce a premature stop codon (nonsense SNP) or have no effect on the protein sequence (synonymous SNP). The promoter region, 5 prime untranslated region (5′UTR), intron and 3′UTR belong to non-coding regions of genes. If the SNPs are located in the 5′UTR, then they may affect the binding activity of transcription factors, thereby leading to the upregulation or downregulation of gene expression level. If the SNPs are located in the 3′UTR, then they may influence microRNA binding to the sequence, thereby affecting gene silencing. In addition, the sequence of the promoter plays a role in initiating gene transcription, which affects the expression of protein. Several studies have determined that several SNPs were related to the antibody response of vaccination (1–3). In our study, we also found that several SNPs were associated with the side effects of COVID-19 vaccines.

In addition, rs3181096 of CD28, as well as rs2227982 and rs36084323 of PDCD1, was associated with side effects of mRNA vaccine in the first and second doses of vaccination. rs733618 of CTLA4, as well as rs1879877, rs3181096 and rs3181098 of CD28, was associated with the side effects of adenoviral vector vaccine in the first and second doses of vaccination. Considering that the components and principles are different in various vaccines, these SNPs may be related to the mechanism by which the components in the vaccine induce side effects. Moreover, these SNPs were associated with several autoimmune diseases and cancer types, which is summarized in **Supplementary Table 5**. Furthermore, rs3181096 of CD28 showed a significant difference in the first and second doses of mRNA vaccines, adenoviral vector vaccine and combination. Finally, rs733618, rs11571316 and rs3087243 of CTLA4 and rs1234314 of TNFSF4 showed great significant difference, although the samples were distributed into the mRNA vaccine group or adenovirus vector vaccine group or their combination. Therefore, these SNPs may play an important role in vaccine-induced immune response.

The CD28 gene is located in the human chromosome 2q33 and the CD28 protein is primarily expressed on the naïve T cell. During T cell activation, CD28 plays an important role in providing a second signal for T cells when interacting with CD80/CD86 (22). Qi et al. showed that rs3181096 was related to the bingeing activity of HNF1_3. When rs3181096 contained C-allele, the transcription level of CD28 would reduce (23). The CTLA4 gene is located in the human chromosome 2q33 and the CTLA4 protein is expressed on activated and regulatory T cells (Tregs). Comparing CD28 with CD80/CD86, CTLA4 was found to play a negative role in T cell activation. Wang et al. showed that rs733618 was involved in regulating the binding activity of NF-1 and c/EBPbeta, and then altering the expression level of CTLA4 (24). These SNPs may modulate the immune response by altering the expression level of CD28 and CTLA4 proteins, leading to vaccine-induced side effects. Therefore, the CD28/CTLA4 pathway is considered as a system that regulates the balance between T cell activation and immune tolerance (22).

The TNFSF4 gene is located in the human chromosome 1q25.1 and composed of 13 exons. It belongs to the TNF superfamily protein and it is the key to the coordination of innate or adaptive immune cells. This protein plays an important role in the life cycle of immune cells, such as differentiation, activation, inhibition, and apoptosis and it is involved in the pathogenesis of various autoimmune and inflammatory diseases (25). Thus, rs1234314 associated with myalgia, joint pain and diarrhoea may be related to the characteristics of this protein.

We observed that SNPs of PDCD1 play a significant role in the side effects induced by mRNA vaccines. In addition, almost all SNPs were associated with fever, chills, headache, myalgia, and joint pain. The PDCD1 gene is located in the human chromosome 2q37. The PD1 molecule will be upregulated on the activated T cell after recognizing the antigen bound to major histocompatibility complex molecules by the T cell receptor. Thus, fever, chills, headache, myalgia and joint pain were due to prolonged antigen expression and PD1 over-expression, leading to excessive local immune response. However, CTLA4 SNPs were primarily associated with injection site discomfort of post-mRNA vaccination. The local immune response induced by vaccines usually results from adjuvants. Adjuvants can directly induce long-term protective immune responses in the host, such as liposomes coated with mRNA or the viral envelope. Adjuvants can help the vaccine induce an immune response and enhance the specificity of the antigen *in vivo* (26). Tritto et al. (7) hypothesized that alum adjuvanticity will delay the absorption of the injected antigen, allowing the antigen to retain at the local injection site in the form of high concentration particles, which will lead to prolonged exposure of antigens to innate immune cells, the release of cytokines and chemokines, and the enhancement of immune response. Then, these cytokines and chemokines will recruit neutrophils, monocytes, and T cells in the blood to the injection site, resulting in redness and swelling. Next, nociception occurs when cytokines, prostaglandins or ATP released by these immune cells interact with nociceptors and the threshold is reached (8). Combining the results of the present study with our previous finding, we found that rs11571315 and rs16840252 of CTLA4 were associated with the adverse reaction of post-haematopoietic stem cell transplantation in patients with acute lymphocytic leukemia (27) and immune thrombocytopenia (28). Moreover, such SNPs were associated with vaccine-induced side effects. Therefore, these two SNPs may be a hub in immune regulation.

This study has also some limitation, including its small sample size, which affect several statistical analyses utilized in within this study was a limitation. Thus, examination of data from a significant number of additional patients is necessary to establish a linkage more definitely between these SNPs and phenotypic responses to SARS-CoV-2 vaccination. In addition, the data are self-reported and the perception of pain and discomfort is different between individuals, which could not be quantified and compared. Moreover, these side effects are general responses to vaccination, not only COVID-19 vaccination.

Therefore, rs3181096 of CD28, rs733618, rs11571316 and rs3087243 of CTLA4, rs2227982 and rs36084323 of PDCD, and rs1234314 of TNFSF4 were associated with mild vaccine side effects. The immune responses originate from T cell activation; therefore, these immune regulatory genes are related to mild symptoms and they may also cause serious side effects. Therefore, the biological functions of SNPs and their effects on T cell activation should be further explored to understand the mechanism of the side effects of vaccination. In clinical application, public concern about the side effects of vaccination could be addressed and the risk of side effects in individuals could be evaluated before vaccination through genetic testing.

## Data availability statement

The original contributions presented in the study are included in the article/**Supplementary Materials**. Further inquiries can be directed to the corresponding author.

## Ethics statement

This study was reviewed and approved by Institutional Review Board (IRB) of Chang Gung Memorial Hospital (CGMH). The approved ID was 202101837B0. The patients/participants provided their written informed consent to participate in this study.

## Author contributions

D-PC: literature review and analysis, statistical analysis and interpretation of data, drafting of manuscript. Y-HW: critical revision of manuscript, literature review. F-PH/W-TL: performed the experiments, analysis and interpretation of data. All authors contributed to the article and approved the submitted version.

## Funding

This study was supported by grants to D-PC from Chang Gung Memorial Hospital (BMRPA81).

## Acknowledgement

We gratefully acknowledged Brian Tang and Enago (Crimson Interactive) for help as regards the poor English language of the manuscript.

## Conflict of interest

The authors declare that the research was conducted in the absence of any commercial or financial relationships that could be construed as a potential conflict of interest.

## Publisher’s note

All claims expressed in this article are solely those of the authors and do not necessarily represent those of their affiliated organizations, or those of the publisher, the editors and the reviewers. Any product that may be evaluated in this article, or claim that may be made by its manufacturer, is not guaranteed or endorsed by the publisher.
