# Supplementary material for: Association between the side effect induced by COVID-19 vaccines and the immune regulatory gene polymorphism
Source: Front Immunol. 2022 Oct 26;13:941497. doi: 10.3389/fimmu.2022.941497 (PMC9643823; doi:10.3389/fimmu.2022.941497)
Supplement: Supplementary file 1 [file Table_1.doc]

Supplementary Material

# Supplementary Table 1. The side effects of first dose of COVID-19 vaccines.

| Types of vaccine | mRNA vaccines | | | | subunit vaccine | | adenoviral vector vaccine | | mixed vaccination | | Total | |
| --- | --- | --- | --- | --- | --- | --- | --- | --- | --- | --- | --- | --- |
|  | Moderna | | BNT | | Medigen | | AZ | |  |  |  |  |
| N | 24 | | 5 | | 5 | | 24 | | 3 | | 61 | |
|  | Y | N | Y | N | Y | N | Y | N | Y | N | Y | N |
| Fever | 29% | 71% | 0% | 100% | 0% | 100% | 71% | 29% | 67% | 33% | 43% | 57% |
| Injection site | 96% | 4% | 80% | 20% | 60% | 40% | 79% | 21% | 100% | 0% | 85% | 15% |
| Chills | 25% | 75% | 40% | 60% | 0% | 100% | 54% | 46% | 100% | 0% | 39% | 61% |
| Diarrhea | 29% | 71% | 0% | 100% | 0% | 100% | 13% | 88% | 67% | 33% | 20% | 80% |
| Headache | 58% | 42% | 0% | 100% | 0% | 100% | 71% | 29% | 100% | 0% | 56% | 44% |
| Myalgia | 33% | 67% | 40% | 60% | 20% | 80% | 67% | 33% | 100% | 0% | 49% | 51% |
| Skin allergy | 21% | 79% | 0% | 100% | 0% | 100% | 4% | 96% | 33% | 67% | 11% | 89% |

N: number of cases; Y: yes; N: no.

**Supplementary Table 2. The side effects of second dose of COVID-19 vaccines.**

| Types of vaccine | mRNA vaccines | | | | subunit vaccine | | adenoviral vector vaccine | | mixed vaccination | | | Total | | |
| --- | --- | --- | --- | --- | --- | --- | --- | --- | --- | --- | --- | --- | --- | --- |
|  | Moderna | | BNT | | Medigen | | AZ | |  |  |  |  |  |  |
| N | 24 | | 5 | | 5 | | 24 | | 3 | | | 61 | | |
|  | Y | N | Y | N | Y | N | Y | N | | Y | N | | Y | N |
| Fever | 63% | 38% | 20% | 80% | 0% | 100% | 29% | 71% | | 33% | 67% | | 39% | 61% |
| Injection site | 100% | 0% | 80% | 20% | 40% | 60% | 42% | 58% | | 67% | 33% | | 69% | 31% |
| Chills | 50% | 50% | 20% | 80% | 20% | 80% | 17% | 83% | | 0% | 100% | | 30% | 70% |
| Diarrhea | 21% | 79% | 0% | 100% | 0% | 100% | 4% | 96% | | 33% | 67% | | 11% | 89% |
| Headache | 67% | 33% | 20% | 80% | 0% | 100% | 8% | 92% | | 67% | 33% | | 34% | 66% |
| Myalgia | 58% | 42% | 60% | 40% | 20% | 80% | 8% | 92% | | 33% | 67% | | 34% | 66% |
| Skin allergy | 17% | 83% | 0% | 100% | 0% | 100% | 0% | 100% | | 33% | 67% | | 8% | 92% |

N: number of cases; Y: yes; N: no.

**Supplementary Table 3. The significant SNPs that were associated with the vaccine side effect with first dose.**

| SNP | Gene position | | No. of patients (%) | | | Model | Logistic regression *P* | Odds ratio | 95% Confidence Interval | |
| --- | --- | --- | --- | --- | --- | --- | --- | --- | --- | --- |
|  |  |  |  |  |  |  |  |  | Lower | Upper |
| Injection site |  |  | |  |  |  |  |  |  |  |
| rs10204525 | PDCD1 | CC | | CT | TT | Additive | 0.017 | - | - | - |
| Yes | intron 4 | 3 (75.0) | | 12 (66.7) | 37(94.9) | CT+CC vs. TT | 0.008 | 0.116 | 0.022 | 0.623 |
| No |  | 1 (25.0) | | 6 (33.3) | 2 (5.1) | CT+TT vs. CC | 0.481 | 0.490 | 0.045 | 5.310 |
| rs2227982 | PDCD1 | AA | | AG | GG | Additive | 0.032 | - | - | - |
| Yes | intron 4 | 19 (100.0) | | 22 (73.3) | 10 (90.9) | AG+AA vs. GG | 1.000 | 0.513 | 0.057 | 4.583 |
| No |  | 0 (0) | | 8 (26.7) | 1 (9.1) | AG+GG vs. AA | 0.046 | - | - | - |
| Chills |  |  | |  |  |  |  |  |  |  |
| rs1879877 | CD28 | GG | | GT | TT | Additive | 0.069 | - | - | - |
| Yes | promoter | 5 (35.7) | | 16 (53.3) | 3 (18.8) | GT+GG vs. TT | 0.043 | 3.957 | 0.988 | 15.850 |
| No |  | 9 (64.3) | | 14 (46.7) | 13 (81.3) | GT+TT vs. GG | 0.709 | 0.789 | 0.228 | 2.730 |
| rs28718975 | CD28 | CC | | CT | TT | Additive | 0.092 | - | - | - |
| Yes | promoter | 1 (33.3) | | 11 (61.1) | 12 (30.8) | CT+CC vs. TT | 0.047 | 3.000 | 0.999 | 9.010 |
| No |  | 2 (66.7) | | 7 (38.9) | 27 (69.2) | CT+TT vs. CC | 1.000 | 0.739 | 0.063 | 8.635 |
| rs28688913 | CD28 | CC | | CT | TT | Additive | 0.048 | - | - | - |
| Yes | promoter | 12 (30.8) | | 11 (61.1) | 0 (0) | CT+CC vs. TT | 0.516 | - | - | - |
| No |  | 27 (69.2) | | 7 (38.9) | 2 (100) | CT+TT vs. CC | 0.071 | 0.364 | 0.119 | 1.107 |
| Diarrhea |  |  | |  |  |  |  |  |  |  |
| rs1879877 | CD28 | GG | | GT | TT | Additive | 0.014 | - | - | - |
| Yes | promoter | 6 (42.9) | | 6 (20.0) | 0 (0) | GT+GG vs. TT | 0.025 | - | - | - |
| No |  | 8 (57.1) | | 9 (80.0) | 16 (100.0) | GT+TT vs. GG | 0.024 | 5.000 | 1.280 | 19.532 |
| rs3181096 | CD28 | CC | | CT | TT | Additive | 0.008 | - | - | - |
| Yes | promoter | 3 (8.6) | | 5 (27.8) | 4 (57.1) | CT+CC vs. TT | 0.025 | 0.133 | 0.025 | 0.712 |
| No |  | 32 (91.4) | | 13 (72.2) | 3 (42.9) | CT+TT vs. CC | 0.009 | 0.167 | 0.040 | 0.702 |
| rs3181098 | CD28 | AA | | AG | GG | Additive | 0.002 | - | - | - |
| Yes | promoter | 4 (66.7) | | 5 (31.3) | 3 (7.9) | AG+AA vs. GG | 0.005 | 8.077 | 1.888 | 34.552 |
| No |  | 2 (33.3) | | 11 (68.8) | 35 (92.1) | AG+GG vs. AA | 0.012 | 11.500 | 1.797 | 73.579 |
| rs5839828 | PDCD1 | G6 | | G6/G7 | G7 | Additive | 0.042 | - | - | - |
| Yes | promoter | 5 (19.2) | | 3 (11.5) | 3 (60.0) | G/del + del/del vs. GG | 0.045 | 0.121 | 0.017 | 0.845 |
| No |  | 21 (80.8) | | 23 (88.5) | 2 (40.0) | G/del + GG vs. del/del | 0.991 | 0.992 | 0.268 | 3.718 |
| Headache |  |  | |  |  |  |  |  |  |  |
| rs733618 | CTLA4 | CC | | CT | TT | Additive | 0.093 | - | - | - |
| Yes | promoter | 3 (37.5) | | 13 (46.4) | 18 (72) | CT+TT vs. CC | 0.447 | 0.426 | 0.092 | 1.971 |
| No |  | 5 (62.5) | | 15 (53.6) | 7 (28) | CT+CC vs. TT | 0.033 | 0.311 | 0.104 | 0.928 |
| rs1879877 | CD28 | GG | | GT | TT | Additive | 0.020 | - | - | - |
| Yes | promoter | 7 (50.0) | | 22 (73.3) | 5 (31.3) | GT+GG vs. TT | 0.017 | 4.253 | 1.247 | 14.511 |
| No |  | 7 (50.0) | | 8 (26.7) | 11 (68.8) | GT+TT vs. GG | 0.565 | 0.704 | 0.212 | 2.338 |
| rs28541784 | CD28 | CC | | CT | TT | Additive | 0.090 | - | - | - |
| Yes | promoter | 19 (48.7) | | 11 (78.6) | 4 (80.0) | CT+CC vs. TT | 0.392 | 0.326 | 0.034 | 3.118 |
| No |  | 20 (51.3) | | 3 (21.4) | 1 (20.0) | CT+TT vs. CC | 0.028 | 0.253 | 0.071 | 0.901 |
| rs201801072 | CD28 | CC | | CT | TT | Additive | 0.010 | 5.526 | 1.382 | 22.104 |
| Yes | promoter | 15 (83.3) | | 0 (0) | 19 (47.5) | CT+CC vs. TT | 0.010 | 5.526 | 1.382 | 22.104 |
| No |  | 3 (16.7) | | 0 (0) | 21 (52.5) | CT+TT vs. CC | 0.010 | 5.526 | 1.382 | 22.104 |
| rs200353921 | CD28 | AA | | AT | TT | Additive | 0.030 | - | - | - |
| Yes | promoter | 12 (80.0) | | 3 (100.0) | 19 (47.5) | AT+AA vs. TT | 0.010 | 5.526 | 1.382 | 22.104 |
| No |  | 3 (20.0) | | 0 (0) | 21 (52.5) | AT+TT vs. AA | 0.051 | 3.818 | 0.942 | 15.473 |
| Myalgia |  |  | |  |  |  |  |  |  |  |
| rs1234314 | TNFSF4 | CC | | CG | GG | Additive | 0.029 | - | - | - |
| Yes | promoter | 5 (71.4) | | 21 (58.3) | 4 (23.5) | CG+CC vs. GG | 0.010 | 4.971 | 1.387 | 17.816 |
| No |  | 2 (28.6) | | 15 (41.7) | 13 (76.5) | CG+GG vs.CC | 0.424 | 2.800 | 0.498 | 15.734 |
| Skin allergy |  |  | |  |  |  |  |  |  |  |
| rs10204525 | PDCD1 | CC | | CT | TT | Additive | 0.107 | - | - | - |
| Yes | intron 4 | 0 (0) | | 0 (0) | 7 (17.9) | CT+CC vs. TT | 0.042 | - | - | - |
| No |  | 4 (100.0) | | 18 (100.0) | 32 (82.1) | CT+TT vs. CC | 1 | - | - | - |

Additive: AA vs. Aa vs. aa

**Supplementary Table 4. The significant SNPs that were associated with the vaccine side effect with second dose.**

| SNP | Gene position | No. of patients (%) | | | Model | Logistic regression *P* | Value | 95% Confidence Interval | |
| --- | --- | --- | --- | --- | --- | --- | --- | --- | --- |
|  |  |  |  |  |  |  |  | Lower | Upper |
| Fever |  |  |  |  |  |  |  |  |  |
| rs3181098 | CD28 | AA | AG | GG | Additive | 0.052 | - | - | - |
| Yes | promoter | 5 (83.3) | 7 (43.8) | 12 (31.6) | AG+AA vs. GG | 0.080 | 2.600 | 0.881 | 7.677 |
| No |  | 1 (16.7) | 9 (56.3) | 26 (68.4) | AG+GG vs. AA | 0.033 | 9.211 | 1.002 | 84.676 |
| rs11571316 | CTLA-4 | AA | AG | GG | Additive | 0.110 | - | - | - |
| Yes | promoter | 2 (50) | 13 (54.2) | 9 (27.3) | AG+AA vs. GG | 0.036 | 3.077 | 1.059 | 8.944 |
| No |  | 2 (50) | 11 (45.8) | 24 (72.7) | AG+GG vs. AA | 0.643 | 1.591 | 0.209 | 12.128 |
| Injection site |  |  |  |  |  |  |  |  |  |
| rs4553808 | CTLA-4 | AA | AG | GG | Additive | 0.011 | - | - | - |
| Yes | promoter | 36 (80) | 7 (43.8) | 0 (0) | AG+AA vs. GG | - | - | - | - |
| No |  | 9 (20) | 9 (56.3) | 0 (0) | AG+GG vs. AA | 0.011 | 5.143 | 1.506 | 17.568 |
| rs62182595 | CTLA-4 | AA | AG | GG | Additive | 0.002 | - | - | - |
| Yes | promoter | 1 (100) | 4 (30.8) | 38 (80.9) | AG+AA vs. GG | 0.002 | 0.132 | 0.035 | 0.489 |
| No |  | 0 (0) | 9 (69.2) | 9 (19.1) | AG+GG vs. AA | 1.000 | - | - | - |
| rs16840252 | CTLA-4 | CC | CT | TT | Additive | 0.011 | - | - | - |
| Yes | promoter | 36 (80) | 7 (43.8) | 0 (0) | CT+TT vs. CC | 0.011 | 5.143 | 1.506 | 17.568 |
| No |  | 9 (20) | 9 (56.3) | 0 (0) | CT+CC vs. TT | - | - | - | - |
| rs10204525 | PDCD1 | CC | CT | TT | Additive | 0.012 | - | - | - |
| Yes | intron 4 | 2 (50) | 8 (44.4) | 32 (82.1) | CT+TT vs. CC | 0.582 | 0.425 | 0.055 | 3.270 |
| No |  | 2 (50) | 10 (55.6) | 7 (17.9) | CT+CC vs. TT | 0.003 | 0.182 | 0.056 | 0.588 |
| Chills |  |  |  |  |  |  |  |  |  |
| rs11571319 | CTLA4 | AA | AG | GG | Additive | 0.126 | - | - | - |
| Yes | 3UTR | 0 (0) | 5 (17.9) | 13 (40.6) | AG+AA vs. GG | 0.046 | 0.304 | 0.092 | 1.005 |
| No |  | 1 (100) | 23 (82.1) | 19 (59.4) | AG+GG vs. AA | 1.000 | - | - | - |
| rs3181096 | CD28 | CC | CT | TT | Additive | 0.135 | - | - | - |
| Yes | promoter | 7 (20) | 8 (44.4) | 3 (42.9) | CT+TT vs. CC | 0.046 | 0.318 | 0.101 | 0.999 |
| No |  | 28 (80) | 10 (55.6) | 4 (57.1) | CT+CC vs. TT | 0.419 | 0.526 | 0.105 | 2.638 |
| rs5839828 | PDCD1 | G6 | G6/G7 | G7 | Additive | 0.010 | - | - | - |
| Yes | promoter | 12 (46.2) | 3 (11.5) | 3 (60) | G/del + del/del vs. GG | 0.312 | 0.270 | 0.041 | 1.784 |
| No |  | 14 (53.8) | 23 (80.5) | 2 (40) | G/del + GG vs. del/del | 0.030 | 3.571 | 1.099 | 11.603 |
| rs41386349 | PDCD1 | AA | AG | GG | Additive | 0.119 |  |  |  |
| Yes | promoter | 2 (50) | 8 (44.4) | 8 (20.5) | AG+AA vs. GG | 0.040 | 3.229 | 1.029 | 10.136 |
| No |  | 2 (50) | 10 (55.6) | 31 (79.5) | AG+GG vs. AA | 0.574 | 2.563 | 0.332 | 19.773 |
| Diarrhea |  |  |  |  |  |  |  |  |  |
| rs4553808 | CTLA-4 | AA | AG | GG | Additive | 0.035 | 4.145 | 1.038 | 16.555 |
| Yes | promoter | 22 (48.9) | 3 (18.8) | 0 (0) | AG+AA vs. GG | - | - | - | - |
| No |  | 23 (51.1) | 13 (81.3) | 0 (0) | AG+GG vs. AA | 0.035 | 4.145 | 1.038 | 16.555 |
| rs62182595 | CTLA-4 | AA | AG | GG | Additive | 0.066 |  |  |  |
| Yes | promoter | 0 (0) | 2 (15.4) | 23 (48.9) | AG+AA vs. GG | 0.021 | 0.174 | 0.035 | 0.864 |
| No |  | 1 (100) | 11 (84.6) | 24 (51.1) | AG+GG vs. AA | 1.000 | 1.029 | 0.973 | 1.087 |
| rs16840252 | CTLA-4 | CC | CT | TT | Additive | 0.035 | 4.145 | 1.038 | 16.555 |
| Yes | promoter | 22 (48.9) | 3 (18.8) | 0 (0) | CT+TT vs. CC | 0.035 | 4.145 | 1.038 | 16.555 |
| No |  | 23 (51.1) | 13 (81.3) | 0 (0) | CT+CC vs. TT | - | - | - | - |
| rs56102377 | CTLA-4 | AA | AG | GG | Additive | 0.037 | 6.800 | 0.910 | 50.810 |
| Yes | exon 4 | 0 (0) | 2 (40) | 5 (8.9) | AG+AA vs. GG | 0.096 | 6.800 | 0.910 | 50.810 |
| No |  | 0 (0) | 3 (60) | 51 (51) | AG+GG vs. AA | - | - | - | - |
| rs200353921 | CD28 | AA | AT | TT | Additive | 0.011 |  |  |  |
| Yes | promoter | 1 (6.7) | 2 (66.7) | 4 (10) | AT+AA vs. TT | 0.665 | 1.800 | 0.359 | 9.037 |
| No |  | 14 (93.3) | 1 (33.3) | 36 (90) | AT+TT vs. AA | 0.664 | 0.440 | 0.049 | 3.993 |
| Headache |  |  |  |  |  |  |  |  |  |
| rs4553808 | CTLA-4 | AA | AG | GG | Additive | 0.032 | 5.115 | 1.038 | 25.218 |
| Yes | promoter | 19 (42.2) | 2 (12.5) | 0 (0) | AG+AA vs. GG | - | - | - | - |
| No |  | 26 (57.8) | 14 (87.5) | 0 (0) | AG+GG vs. AA | 0.032 | 5.115 | 1.038 | 25.218 |
| rs11571316 | CTLA-4 | AA | AG | GG | Additive | 0.027 | - | - | - |
| Yes | promoter | 3 (75) | 4 (16.7) | 14 (42.4) | AG+AA vs. GG | 0.153 | 0.452 | 0.151 | 1.358 |
| No |  | 1 (25) | 20 (83.3) | 19 (57.6) | AG+GG vs. AA | 0.113 | 6.500 | 0.632 | 66.878 |
| rs62182595 | CTLA-4 | AA | AG | GG | Additive | 0.049 | - | - | - |
| Yes | promoter | 0 (0) | 1 (7.7) | 20 (42.6) | AG+AA vs. GG | 0.022 | 0.104 | 0.013 | 0.860 |
| No |  | 1 (100) | 12 (92.3) | 27 (57.4) | AG+GG vs. AA | 1.000 | - | - | - |
| rs16840252 | CTLA-4 | CC | CT | TT | Additive | 0.032 | 5.115 | 1.038 | 25.218 |
| Yes | promoter | 19 (42.2) | 2 (12.5) | 0 (0) | CT+TT vs. CC | 0.032 | 5.115 | 1.038 | 25.218 |
| No |  | 26 (57.8) | 14 (87.5) | 0 (0) | CT+CC vs. TT | - | - | - | - |
| rs3087243 | CTLA4 | AA | AG | GG | Additive | 0.062 | - | - | - |
| Yes | 3UTR | 2 (50) | 12 (50) | 7 (21.2) | AG+AA vs. GG | 0.018 | 3.714 | 1.217 | 11.338 |
| No |  | 2 (50) | 12 (50) | 26 (78.8) | AG+GG vs. AA | 0.602 | 2.000 | 0.261 | 15.318 |
| rs3181096 | CD28 | CC | CT | TT | Additive | 0.002 | - | - | - |
| Yes | promoter | 7 (20) | 8 (44.4) | 6 (85.7) | CT+TT vs. CC | 0.004 | 0.196 | 0.063 | 0.617 |
| No |  | 28 (80) | 10 (55.6) | 1 (14.3) | CT+CC vs. TT | 0.006 | 0.066 | 0.007 | 0.594 |
| rs3181097 | CD28 | AA | AG | GG | Additive | 0.022 | - | - | - |
| Yes | promoter | 2 (18.2) | 6 (23.1) | 13 (56.5) | AG+AA vs. GG | 0.006 | 0.212 | 0.068 | 0.661 |
| No |  | 9 (81.8) | 20 (76.9) | 10 (43.5) | AG+GG vs. AA | 0.299 | 0.351 | 0.068 | 1.802 |
| rs3181098 | CD28 | AA | AG | GG | Additive | 0.004 | - | - | - |
| Yes | promoter | 5 (5) | 8 (50) | 8 (21.1) | AG+AA vs. GG | 0.003 | 5.417 | 1.709 | 17.167 |
| No |  | 1 (16.7) | 8 (50) | 30 (78.9) | AG+GG vs. AA | 0.017 | 11.875 | 1.283 | 109.893 |
| rs200353921 | CD28 | AA | AT | TT | Additive | 0.030 | - | - | - |
| Yes | promoter | 3 (20) | 3 (100) | 15 (37.5) | AT+AA vs. TT | 0.760 | 0.833 | 0.259 | 2.686 |
| No |  | 12 (80) | 0 (0) | 25 (62.5) | AT+TT vs. AA | 0.129 | 0.347 | 0.085 | 1.412 |
| rs10204525 | PDCD1 | CC | CT | TT | Additive | 0.127 | - | - | - |
| Yes | intron 4 | 1 (25) | 3 (16.7) | 17 (43.6) | CT+TT vs. CC | 1.000 | 0.617 | 0.060 | 6.323 |
| No |  | 3 (75) | 15 (83.3) | 22 (56.4) | CT+CC vs. TT | 0.045 | 0.288 | 0.082 | 1.009 |
| Myalgia |  |  |  |  |  |  |  |  |  |
| rs1879877 | CD28 | GG | GT | TT | Additive | 0.028 | - | - | - |
| Yes | promoter | 9 (64.3) | 7 (23.3) | 5 (31.3) | GT+GG vs. TT | 0.713 | 1.257 | 0.370 | 4.269 |
| No |  | 5 (35.7) | 23 (76.7) | 11 (68.8) | GT+TT vs. GG | 0.012 | 5.100 | 1.424 | 18.270 |
| rs200353921 | CD28 | AA | AT | TT | Additive | 0.029 | - | - | - |
| Yes | promoter | 3 (20) | 3 (100) | 14 (35) | AT+AA vs. TT | 0.902 | 0.929 | 0.287 | 3.009 |
| No |  | 12 (80) | 0 (0) | 26 (65) | AT+TT vs. AA | 0.171 | 0.382 | 0.094 | 1.558 |
| rs10204525 | PDCD1 | CC | CT | TT | Additive | 0.030 | - | - | - |
| Yes | intron 4 | 0 (0) | 3 (16.7) | 18 (46.2) | CT+TT vs. CC | 0.289 | - | - | - |
| No |  | 4 (100) | 15 (83.3) | 21 (53.8) | CT+CC vs. TT | 0.010 | 0.184 | 0.047 | 0.725 |
| Skin allergy |  |  |  |  |  |  |  |  |  |
| rs11571315 | CTLA-4 | CC | CT | TT | Additive | 0.063 | - | - | - |
| Yes | promoter | 0 (0) | 1 (3.8) | 5 (20.8) | CT+TT vs. CC | 0.580 | - | - | - |
| No |  | 11 (100) | 25 (96.2) | 19 (79.2) | CT+CC vs. TT | 0.031 | 0.106 | 0.011 | 0.970 |
| rs733618 | CTLA-4 | CC | CT | TT | Additive | 0.016 | - | - | - |
| Yes | promoter | 3 (37.5) | 1 (3.6) | 2 (8) | CT+TT vs. CC | 0.025 | 10.00 | 1.579 | 63.316 |
| No |  | 5 (62.5) | 27 (94.4) | 23 (92) | CT+CC vs. TT | 1.000 | 1.438 | 0.242 | 8.524 |
| rs11571316 | CTLA-4 | AA | AG | GG | Additive | 0.059 | - | - | - |
| Yes | promoter | 0 (0) | 0 (0) | 6 (18.2) | AG+AA vs. GG | 0.027 | - | - | - |
| No |  | 4 (100) | 24 (100) | 27 (81.8) | AG+GG vs. AA | 1.000 | - | - | - |
| rs1581575882 | CTLA4 | AA | AG | GG | Additive | 0.001 | - | - | - |
| Yes | 3UTR | 0 (0) | 4 (6.7) | 1 (100) | AG+AA vs. GG | 0.082 | - | - | - |
| No |  | 0 (0) | 56 (93.3) | 0 (0) | AG+GG vs. AA | - | - | - | - |

Additive: AA vs. Aa vs. aa

**Supplementary Table 5. Summarized the SNPs of vaccine-induced side effects and the diseases related to these significant SNPs showed in literature.**

| **SNP** | **Combination** | | **mRNA**  **vaccines** | | **adenoviral vector vaccine** | | **disease** | **reference** |
| --- | --- | --- | --- | --- | --- | --- | --- | --- |
|  | 1st | 2nd | 1st | 2nd | 1st | 2nd |  |  |
| **CTLA4** |  |  |  |  |  |  |  |  |
| rs11571315 | 🗸 |  |  | 🗸 | 🗸 | 🗸 | transfusion reaction | [1] |
|  |  |  |  |  |  |  | Polycystic ovary syndrome | [2] |
| rs11571316 | 🗸 |  |  |  |  |  | urinary schistosomiasis | [3] |
|  |  |  |  |  |  |  | asthma | [4] |
|  |  |  |  |  |  |  | cervical cancer | [5] |
| rs733618 | 🗸 | 🗸 | 🗸 | 🗸 | 🗸 | 🗸 | urinary schistosomiasis | [3] |
|  |  |  |  |  |  |  | lymphatic filariasis | [6] |
|  |  |  |  |  |  |  | systemic lupus erythematosus | [7] |
|  |  |  |  |  |  |  | treatment outcomes of patients with multiple myeloma receiving bortezomib-based regimens | [8] |
|  |  |  |  |  |  |  | myasthenia gravis | [9] |
|  |  |  |  |  |  |  | sepsis | [10] |
|  |  |  |  |  |  |  | Graves’ disease | [11] |
| rs231775 | 🗸 |  |  | 🗸 | 🗸 |  | type 1 diabetes | [12] [13] [14] |
|  |  |  |  |  |  |  | Graves’ disease | [15] [16] |
|  |  |  |  |  |  |  | thyroid carcinoma | [17] |
|  |  |  |  |  |  |  | systemic lupus erythematosus | [18] |
|  |  |  |  |  |  |  | autoimmune Disease | [19] |
|  |  |  |  |  |  |  | polyarticular juvenile idiopathic arthritis | [20] |
|  |  |  |  |  |  |  | urinary schistosomiasis | [3] |
|  |  |  |  |  |  |  | cancers | [21] |
|  |  |  |  |  |  |  | sepsis | [10] |
| rs3087243 | 🗸 | 🗸 |  | 🗸 | 🗸 | 🗸 | sepsis | [10] |
|  |  |  |  |  |  |  | type 1 diabetes | [12] |
|  |  |  |  |  |  |  | allogeneic hematopoietic stem cell transplantation | [22] |
|  |  |  |  |  |  |  | rheumatoid arthritis | [23] |
| rs980967681 |  |  | 🗸 |  | 🗸 | 🗸 |  |  |
| **CD28** |  |  |  |  |  |  |  |  |
| rs1879877 | 🗸 | 🗸 |  |  | 🗸 | 🗸 | type 1 diabetes | [24] |
| rs3181097 |  | 🗸 |  | 🗸 | 🗸 | 🗸 | transfusion reaction | [26] |
| rs3181096 | 🗸 | 🗸 | 🗸 | 🗸 | 🗸 | 🗸 | type 1 diabetes | [27] [24] |
| rs3181098 | 🗸 | 🗸 | 🗸 | 🗸 | 🗸 | 🗸 | malignant melanoma | [28] |
|  |  |  |  |  |  |  | recurrent spontaneous abortion | [29] |
|  |  |  |  |  |  |  | renal cell carcinoma | [25] |
| rs28718975 | 🗸 |  |  |  | 🗸 |  |  |  |
| rs28688913 | 🗸 |  | 🗸 |  |  |  |  |  |
| rs28541784 | 🗸 |  |  |  |  |  |  |  |
| rs201801072 | 🗸 |  |  |  |  |  |  |  |
| rs200353921 | 🗸 | 🗸 | 🗸 | 🗸 |  |  |  |  |
| **PDCD1** |  |  |  |  |  |  |  |  |
| rs2227981 | 🗸 | 🗸 | 🗸 | 🗸 |  |  | prognosis of metastatic melanoma patient treated with anti-PD-1 monotherapy | [30] |
|  |  |  |  |  |  |  | breast cancer | [31] |
|  |  |  |  |  |  |  | human T-cell leukemia virus type 1 | [32] |
| rs10204525 | 🗸 | 🗸 |  | 🗸 | 🗸 |  | polyarticular juvenile idiopathic arthritis | [20] |
|  |  |  |  |  |  |  | viral load in HIV-1 infected patients | [33] |
|  |  |  |  |  |  |  | acute anterior uveitis | [34] |
|  |  |  |  |  |  |  | human T-cell leukemia virus type 1 | [32] |
| rs6705653 | 🗸 | 🗸 | 🗸 | 🗸 |  |  | Systemic lupus erythematosus | [35] |
| rs2227982 | 🗸 | 🗸 | 🗸 | 🗸 |  |  | adverse events of patients with non-small cell lung cancer treated by nivolumab | [36] |
|  |  |  |  |  |  |  | breast cancer | [31] |
|  |  |  |  |  |  |  | acute anterior uveitis | [34] |
|  |  |  |  |  |  |  | multiple myeloma | [37] |
| rs41386349 | 🗸 | 🗸 | 🗸 | 🗸 |  |  | breast cancer | [31] |
| rs5839828 | 🗸 | 🗸 | 🗸 | 🗸 |  |  | colorectal cancer | [38] |
| rs36084323 |  | 🗸 | 🗸 | 🗸 |  |  | cervical cancer | [39] |
|  |  |  |  |  |  |  | primary immune thrombocytopenia | [40] |
|  |  |  |  |  |  |  | colorectal cancer | [41] |
|  |  |  |  |  |  |  | non-small cell lung cancer | [42] |
| **TNFSF4** |  |  |  |  |  |  |  |  |
| rs1234314 | 🗸 |  | 🗸 | 🗸 | 🗸 |  | children asthma and allergic rhinitis | [43] |
| rs45454293 |  | 🗸 |  |  |  | 🗸 | large artery atherosclerosis | [44] |

1. Wen YH, Lin WT, Wang WT, Chiueh TS, Chen DP. Association of CTLA4 gene polymorphism with transfusion reaction after infusion of leukoreduced blood xomponent. *J Clin Med* (2019) 8(11):1961. [doi:10.3390/jcm8111961](https://doi.org/10.3390/jcm8111961).
2. Rawaa Abdul-Ameer Abdul-Jabbar. Significance of CTL4 Gene Polymorphisms in Susceptibility to Polycystic Ovary Syndrome of Iraqi Women. *Annals of RSCB* (2021) 25(1):6624-6632.
3. Idris ZM, Yazdanbakhsh M, Adegnika AA, Lell B, Issifou S, Noordin R. A pilot study on cytotoxic T lymphocyte-4 gene polymorphisms in urinary schistosomiasis. *Genet Test Mol Biomarkers* (2012) 16(6):488-92.  [doi:10.1089/gtmb.2011.0209](https://doi.org/10.1089/gtmb.2011.0209).
4. Choi H, Tabashidze N, Rossner P Jr, Dostal M, Pastorkova A, Kong SW, et al. Altered vulnerability to asthma at various levels of ambient Benzo[a]Pyrene by CTLA4, STAT4 and CYP2E1 polymorphisms. Environ Pollut (2017) 231(Pt 1):1134-1144. doi:10.1016/j.envpol.2017.07.057.
5. Jiang L, Luo RY, Zhang W, Wang LR, Wang F, Cheng YX. Single nucleotide polymorphisms of CTLA4 gene and their association with human cervical cancer. *Zhonghua Yi Xue Yi Chuan Xue Za Zhi* (2011) 28(3):313-7. doi:10.3760/cma.j.issn.1003-9406.2011.03.017
6. Idris ZM, Miswan N, Muhi J, Mohd TA, Kun JF, Noordin R. Association of CTLA4 gene polymorphisms with lymphatic filariasis in an East Malaysian population. *Hum Immunol* (2011) 72(7):607-12. doi: 10.1016/j.humimm.2011.03.017.
7. Liu J, Zhang H. -1722T/C polymorphism (rs733618) of CTLA-4 significantly associated with systemic lupus erythematosus (SLE): a comprehensive meta-analysis. *Hum Immunol* (2013) 74(3):341-7. doi: 10.1016/j.humimm.2012.12.009.
8. Qin XY, Lu J, Li GX, Wen L, Liu Y, Xu LP, et al. CTLA-4 polymorphisms are associated with treatment outcomes of patients with multiple myeloma receiving bortezomib-based regimens. *Ann Hematol* (2018) 97(3):485-495. doi: 10.1007/s00277-017-3203-7.
9. Li HF, Hong Y, Zhang X, Xie Y, Skeie GO, et al. Gene polymorphisms for both auto-antigen and immune-modulating proteins are associated with the susceptibility of autoimmune myasthenia gravis. *Mol Neurobiol* (2017) 54(6):4771-4780. doi: 10.1007/s12035-016-0024-y.
10. Mewes C, Büttner B, Hinz J, Alpert A, Popov AF, Ghadimi M, et al. CTLA-4 genetic variants predict survival in patients with sepsis. *J Clin Med* (2019) 8(1):70. doi: 10.3390/jcm8010070.
11. Chen DP, Chu YC, Wen YH, Lin WT, Hour AL, Wang WT. investigation of the correlation between Graves' ophthalmopathy and CTLA4 gene polymorphism. *J Clin Med* (2019) 8(11):1842. doi: 10.3390/jcm8111842.
12. Sharma C, R Ali B, Osman W, Afandi B, Aburawi EH, Beshyah SA, et al. Association of variants in PTPN22, CTLA-4, IL2-RA, and INS genes with type 1 diabetes in Emiratis. *Ann Hum Genet* (2021) 85(2):48-57.  [doi:10.1111/ahg.12406](https://doi.org/10.1111/ahg.12406).
13. Borysewicz-Sańczyk H, Sawicka B, Wawrusiewicz-Kurylonek N, Głowińska-Olszewska B, Kadłubiska A, Gościk J, et al. Genetic association study of IL2RA, IFIH1, and CTLA-4 polymorphisms with autoimmune thyroid diseases and type 1 diabetes. *Front Pediatr* (2020) 8:481. doi:10.3389/fped.2020.00481.
14. Chen Y, Chen S, Gu Y, Feng Y, Shi Y, Fu Q, et al. CTLA-4 +49 G/A, a functional T1D risk SNP, affects CTLA-4 level in Treg subsets and IA-2A positivity, but not beta-cell function. *Sci Rep* (2018) 8(1):10074.  [doi:10.1038/s41598-018-28423-9](https://doi.org/10.1038/s41598-018-28423-9).
15. Chen PL, Fann CS, Chang CC, Wu IL, Chiu WY, Lin CY, et al. Family-based association study of cytotoxic T-lymphocyte antigen-4 with susceptibility to Graves' disease in Han population of Taiwan. *Genes Immun* (2008) 9(2):87-92. doi:10.1038/sj.gene. 6364445.
16. Gu LQ, Zhu W, Zhao SX, Zhao L, Zhang MJ, Cui B, et al. Clinical associations of the genetic variants of CTLA-4, Tg, TSHR, PTPN22, PTPN12 and FCRL3 in patients with Graves' disease. *Clin Endocrinol* (Oxf) (2010) 72(2):248-55. [doi:10.1111/j.1365-2265.2009.03617.x](https://doi.org/10.1111/j.1365-2265.2009.03617.x).
17. Abtahi S, Izadi Jahromi F, Dabbaghmanesh MH, Malekzadeh M, Ghaderi A. Association between CTLA-4 + 49A > G and - 318C > T single-nucleotide polymorphisms and susceptibility to thyroid neoplasm. *Endocrine* (2018) 62(1):159-165. doi:10.1007/ s12020-018-1663-8.
18. Devaraju P, Gulati R, Singh BK, Mithun CB, Negi VS. The CTLA4 +49 A/G (rs231775) polymorphism influences susceptibility to SLE in South Indian Tamils. *Tissue Antigens* (2014) 83(6):418-21.  [doi:10.1111/tan.12363](https://doi.org/10.1111/tan.12363).
19. Wang K, Zhu Q, Lu Y, Lu H, Zhang F, Wang X, et al. CTLA-4 +49 G/A polymorphism confers autoimmune disease risk: an updated meta-analysis. *Genet Test Mol Biomarkers* (2017) 21(4):222-227. [doi:10.1089/gtmb.2016.0335](https://doi.org/10.1089/gtmb.2016.0335).
20. Ali MA, Abdelaziz A, Ali M, Abonar A, Hanafy M, Hussein H, et al. PADI4 (rs2240340), PDCD1 (rs10204525), and CTLA4 (231775) gene polymorphisms and polyarticular juvenile idiopathic arthritis. *Br J Biomed Sci* (2020) 77(3):123-128. doi:10.1080/ 09674845.2020.
21. Fang M, Huang W, Mo D, Zhao W, Huang R. Association of five SNPs in cytotoxic T-lymphocyte antigen 4 and cancer susceptibility: evidence from 67 studies. *Cell Physiol Biochem* (2018) 47(1):414-427. doi: 10.1159/000489953.
22. Najafi A, Alizadeh-Navaei R, Rahimi S, Valadan R, Tehrani M. Genetic polymorphisms of cytotoxic T-lymphocyte antigen 4 (CTLA-4) and clinical outcomes post-allogeneic hematopoietic stem cell transplantation: a systematic review and meta-analysis. *Clin Transplant* (2021) 35(8):e14364. doi: 10.1111/ctr.14364.
23. Aslam MM, Jalil F, John P, Fan KH, Bhatti A, Feingold E, et al. A sequencing study of CTLA4 in Pakistani rheumatoid arthritis cases. *PLoS One* (2020) 15(9):e0239426. doi: 10.1371/journal.pone.0239426.
24. Zouidi F, Stayoussef M, Bouzid D, Fourati H, Abida O, Ayed MB, et al. Contribution of PTPN22, CD28, CTLA-4 and ZAP-70 variants to the risk of type 1 diabetes in Tunisians. *Gene* (2014) 533(1):420-6. doi: 10.1016/j.gene.2013.09.112.
25. Tupikowski K, Partyka A, Kolodziej A, Dembowski J, Debinski P, Halon A, et al. CTLA-4 and CD28 genes' polymorphisms and renal cell carcinoma susceptibility in the Polish population--a prospective study. *Tissue Antigens* (2015) 86(5):353-61. doi: 10.1111/tan.12671.
26. Wen YH, Lin WT, Wang WT, Chen DP. CD28 gene polymorphisms in the promoter region are associated with transfusion reactions: a functional study. *J Clin Med* (2021) 20;10(4):871. doi:10.3390/jcm10040871.
27. Ferjeni Z, Bouzid D, Fourati H, Stayoussef M, Abida O, Kammoun T, et al. Association of TCR/CD3, PTPN22, CD28 and ZAP70 gene polymorphisms with type 1 diabetes risk in Tunisian population: family based association study. Immunol Lett (2015) 163(1):1-7. doi:10.1016/j.imlet.2014.11.005.
28. Bouwhuis MG, Gast A, Figl A, Eggermont AM, Hemminki K, Schadendorf D, et al. Polymorphisms in the CD28/CTLA4/ICOS genes: role in malignant melanoma susceptibility and prognosis? *Cancer Immunol Immunother* (2010) 59(2):303-12. doi:10.1007/ s00262-009-0751-2.
29. Wang G, Sun J. Interactive Effects of Snps Located Within CD28/B7Pathway and Environment on Susceptibility to Recurrent Spontaneous Abortion. *Cell Physiol Biochem* (2017) 43(6):2185-2199. doi:10.1159/000484297.
30. de With M, Hurkmans DP, Oomen-de Hoop E, Lalouti A, Bins S, El Bouazzaoui S, et al. Germline variation in PDCD1 is associated with overall survival in patients with metastatic melanoma treated with anti-PD-1 monotherapy. *Cancers (Basel)* (2021) 18;13(6):1370. doi:10.3390/cancers13061370.
31. Karami S, Sattarifard H, Kiumarsi M, Sarabandi S, Taheri M, Hashemi M, et al. Evaluating the possible association between PD-1 (rs11568821, rs2227981, rs2227982) and PD-L1 (rs4143815, rs2890658) polymorphisms and susceptibility to breast cancer in a sample of southeast Iranian women. *Asian Pac J Cancer Prev* (2020) 21(10):3115-3123. doi:10.31557/APJCP.2020.21.10.3115.
32. Hezave YA, Sharifi Z, Ranjbar Kermani F, Shahabi M. The association of polymorphisms (rs2227981 and rs10204525) of PDCD1 gene with susceptibility to human T-cell leukemia virus type 1. *Microb Pathog* (2021) 158:105049. doi: 10.1016/j.micpath.2021.105049.
33. Baba H, Kettani A, Bouqdayr M, Ouladlahsen A, Bensghir R, Marih L, et al. Programmed cell death-1 single-nucleotide polymorphism rs10204525 is associated with human immunodeficiency virus type 1 RNA viral load in HIV-1-infected Moroccan subjects. *Med Microbiol Immunol* (2021) 210(4):187-196. doi:10.1007/s00430-021-00712-7.
34. Li Y, Hong M, Huang X, Zhong L, Gu Y, Wang D, et al. PD-1 polymorphisms are associated with susceptibility of acute anterior uveitis in Chinese population. *DNA Cell Biol* (2019) 38(2):121-128. doi: 10.1089/dna.2018.4417.
35. Khanjari Y, Oladnabi M, Abdollahi N, Heidari A, Mohammadi S, Tabarraei A. Variants in ntron 4 of PD-1 gene are associated with the susceptibility to SLE in an Iranian population. *Iran J Immunol* (2020) 17(3):204-214. doi:10.22034/iji.2020.83046.1610.
36. Bins S, Basak EA, El Bouazzaoui S, Koolen SLW, Oomen-de Hoop E, van der Leest CH, et al. Association between single-nucleotide polymorphisms and adverse events in nivolumab-treated non-small cell lung cancer patients. *Br J Cancer* (2018) 118(10):1296-1301. doi:10.1038/s41416-018-0074-1.
37. Kasamatsu T, Awata M, Ishihara R, Murakami Y, Gotoh N, Matsumoto M, et al. PDCD1 and PDCD1LG1 polymorphisms affect the susceptibility to multiple myeloma. *Clin Exp Med* (2020) 20(1):51-62. doi: 10.1007/s10238-019-00585-4.
38. Ge J, Zhu L, Zhou J, Li G, Li Y, Li S, et al. Association between co-inhibitory molecule gene tagging single nucleotide polymorphisms and the risk of colorectal cancer in Chinese. *J Cancer Res Clin Oncol* (2015) 141(9):1533-44. [doi:10.31557/10.1007/s00432-015-1915-4](https://doi.org/10.31557/10.1007/s00432-015-1915-4)
39. da Silva MC, Medeiros FS, da Silva NCH, Paiva LA, Gomes FODS, Costa E Silva M, et al. Increased PD-1 level in severe cervical injury is associated with the rare programmed cell death 1 (PDCD1) rs36084323 A allele in a dominant model. *Front Cell Infect Microbiol* (2021) 11:587932. doi: 10.3389/fcimb.2021.587932.
40. Wang S, Zhang X, Leng S, Xu Q, Sheng Z, Zhang Y, et al. Immune checkpoint-related gene polymorphisms are associated with primary immune thrombocytopenia. *Front Immunol* (2021) 11:615941. doi: 10.3389/fimmu.2020.615941.
41. Cevik M, Namal E, Iner-Koksal U, Dinc-Sener N, Karaalp A, Ciftci C, et al. Association of PD-1 and PDL-1 gene polymorphisms with colorectal cancer risk and prognosis. *Mol Biol Rep* (2022) 49(3):1827-1836. doi: 10.1007/s11033-021-06992-9.
42. Sasaki H, Tatemaysu T, Okuda K, Moriyama S, Yano M, Fujii Y. PD-1 gene promoter polymorphisms correlate with a poor prognosis in non-small cell lung cancer. *Mol Clin Oncol* (2014) 2(6):1035-1042. doi: 10.3892/mco.2014.358.
43. Liu Y, Ke X, Kang HY, Wang XQ, Shen Y, Hong SL. Genetic risk of TNFSF4 and FAM167A-BLK polymorphisms in children with asthma and allergic rhinitis in a Han Chinese population. *J Asthma* 2016 53(6):567-75. doi: 10.3109/02770903.2015.1108437.
44. Jiang Y, Liu X, Du Y, Zhou S. rs1234313 and rs45454293 are risk factors of cerebral arterial thrombosis, large artery atherosclerosis, and carotid plaque in the Han Chinese population: a case-control study. *BMC Neurol* (2019) 19(1):31. doi: 10.1186/s12883-019-1259-9.
